# Suggested Modifications to the Management of Patients With Breast Cancer During the COVID-19 Pandemic: Web-Based Survey Study

**DOI:** 10.2196/27073

**Published:** 2021-11-15

**Authors:** Shereef Elsamany, Mohamed Elbaiomy, Ahmed Zeeneldin, Emad Tashkandi, Fayza Hassanin, Nafisa Abdelhafeez, Humaid O Al-Shamsi, Nedal Bukhari, Omima Elemam

**Affiliations:** 1 Oncology Center King Abdullah Medical City Makkah Saudi Arabia; 2 Oncology Center Mansoura University Mansoura Egypt; 3 National Cancer Institute Cairo University Cairo Egypt; 4 College of Medicine Umm AlQura University Makkah Saudi Arabia; 5 Oncology Center King Abdulaziz Medical City Riyadh Saudi Arabia; 6 King Saud bin Abdulaziz University for Health Sciences Riyadh Saudi Arabia; 7 Department of Oncology Alzahra Hospital Dubai United Arab Emirates; 8 University of Sharjah Sharjah United Arab Emirates; 9 Department of Medical Oncology King Fahad Specialist Hospital Dammam Saudi Arabia; 10 Department of Internal Medicine Imam Abdulrahman Bin Faisal University Dammam Saudi Arabia

**Keywords:** breast cancer, COVID-19, pandemic, web-based survey, treatment modification, oncology, treatment, modification, risk, infection

## Abstract

**Background:**

Management of patients with cancer in the current era of the COVID-19 pandemic poses a significant challenge to health care systems. Breast cancer is the most common cancer internationally. Breast cancer is a disease that involves surgery, chemotherapy, hormonal therapy, targeted therapy, radiotherapy, and, more recently, immunotherapy in its management plan. The immune system requires months to recover from these medications, and this condition is even worse in patients with metastatic breast cancer who need ongoing treatment with these drugs. Some of these drugs, such as inhibitors of cyclin-dependent kinases 4 and 6, can cause rare but life-threating lung inflammation. Patients with breast cancer who have metastatic disease to the lungs can experience deterioration of disease symptoms with COVID-19 infection. Oncologists treating patients with breast cancer are facing a difficult situation regarding treatment choice. The impact that COVID-19 has had on breast cancer care is unknown, including how to provide the best care possible without compromising patient and community safety.

**Objective:**

The aim of this study was to explore the views of oncologists regarding the management of patients with breast cancer during the COVID-19 pandemic.

**Methods:**

A web-based SurveyMonkey questionnaire was submitted to licensed oncologists involved in breast cancer management in Saudi Arabia, Egypt, and United Arab Emirates. The survey focused on characteristics of the participants, infection risk among patients with cancer, and possible treatment modifications related to different types of breast cancer.

**Results:**

The survey was completed by 82 participants. For early hormone receptor (HR)–positive, human epidermal growth factor receptor 2 (HER2)–negative breast cancer, 61 of the 82 participants (74%) supported using neoadjuvant hormonal therapy in selected patients, and 58% (48/82) preferred giving 6 over 8 cycles of adjuvant chemotherapy when indicated. Only 43% (35/82) preferred inhibitors of cyclin-dependent kinases 4 and 6 with hormonal therapy as the first-line treatment in all patients with metastatic HR-positive disease. A total of 55 of the 82 participants (67%) supported using adjuvant trastuzumab for 6 instead of 12 months in selected patients with HER2-positive breast cancer. For metastatic HER2-positive, HR-positive breast cancer, 80% of participants (66/82) supported the use of hormonal therapy with dual anti-HER2 blockade in selected patients. The preferred choice of first-line treatment in metastatic triple negative patients with *BRCA* mutation and programmed cell death 1 ligand 1 (*PD-L1*) <1% was poly(adenosine diphosphate–ribose) polymerase inhibitor according to 41% (34/82) of the participants, and atezolizumab with nab-paclitaxel was preferred for *PD-L1* >1% according to 71% (58/82) of the participants.

**Conclusions:**

Several modifications in breast cancer management were supported by the survey participants. These modifications need to be discussed on a local basis, taking into account the local infrastructure and available resources.

## Introduction

Management of patients with cancer in the current era of the COVID-19 pandemic poses a significant challenge to health care systems [1]. However, it is mandatory to maintain the required level of care of patients with cancer while taking the necessary precautions to maintain the safety of both patients and health care professionals (HCPs) [2-4]. Nevertheless, certain modifications of medical management of patients with cancer, including surgical approaches, locoregional therapies, and systemic therapies, in addition to changes in treatment and follow-up schedules are required to maintain the balance between the care and safety of patients. In addition, setting priorities for medical care may be required when the available health services are insufficient for the number of patients who need care [1]. Patients with cancer can be considered a heterogeneous group of patients with different presentations, stages at diagnosis, tumor burdens, and therapeutic modalities with associated adverse events and related immune suppression. Thus, patients with cancer may have variable risk of COVID-19–related complications [5].

Patients with breast cancer, at least in part, are more vulnerable to COVID-19 infection due to a variety of reasons, including myelosuppression produced by chemotherapy given in (neo)adjuvant or metastatic settings [6], inhibitors of cyclin-dependent kinases 4 and 6 (CDK4/6) [7-9], and palliative radiotherapy to the spine or pelvis. In addition, myelosuppression can be secondary to bone marrow infiltration by metastatic tumor cells. Different scientific and medical societies have released suggestions and recommendations that address possible treatment modifications and precautions in the management of patients with cancer in the era of the COVID-19 pandemic, such as the European Society of Medical Oncology (ESMO) [10], American College of Surgeons [11], and National Comprehensive Cancer Network [12].

The main theme of these expert opinion–based recommendations focuses on reducing the probability or duration of neutropenia, reducing the frequency of hospital visits and stays, and avoiding medications that may be dangerous to use during the current COVID-19 pandemic. For example, the ESMO recommendations dissect the priority of the management of patients with breast cancer into low, medium, and high priorities for medical care [10]. Similarly, Cancer Care Ontario reported different priorities for medical care of patients with cancer using variable therapeutic modalities, including surgery, radiotherapy, systemic therapy, and palliative care [13]. Furthermore, the American College of Surgeons provided pragmatic suggestions for triaging patients for surgical management based on the volume of COVID-19 cases, available intensive care unit (ICU) capacity, available hospital resources, and degree of urgency of surgical management [11].

Therefore, during the COVID-19 pandemic, it may be necessary to reconsider the risk to benefit ratio of different treatment modalities to select the best therapeutic strategy for each patient. Therefore, discussion in multidisciplinary tumor boards and assessment of available hospital facilities are critically important. Moreover, it is crucial to check the response of practicing oncologists to these recommendations of therapeutic modifications and determine whether they are being adopted in real practice. In this survey study, we will explore the views of oncologists treating patients with breast cancer on possible modifications in breast cancer management in the current period of the COVID-19 pandemic. This survey will include suggested modifications by key medical societies in different subtypes of breast cancer, focusing mainly on systemic therapy. In addition, the survey may help fill the gap between guidelines recommended by scientific societies in the COVID-19 era and what is actually occurring in everyday clinical practice in three Middle Eastern countries. These countries have different health care systems, economic resources, and patient volumes. This study will shed light on how these potential modifications can actually guide oncology practice in the current era.

## Methods

### Development of the Instrument

We generated our survey instrument using rigorous survey development and testing methods [14]. Items were selected based on a literature review, emails, and telephone correspondence. Three experts in the field of breast cancer from King Abdullah Medical City, Saudi Arabia, extensively discussed the topic and reviewed items until no further questions were raised. Items were nominated and then ranked by expert breast oncologists to reach a consensus on the selected items. Further review was performed to eliminate redundant items using binary responses (exclude and include). Fuzzy logic was applied to check the consensus among the experts in a more robust way than in the traditional method [14].

During construction of the survey, we grouped the items into the domains we wanted to explore and then refined the questions [15]. The self-administered survey consisted of 25 items that focused on 5 domains: characteristics of participants; COVID-19 infection risk among patients with cancer/need for treatment modifications; and possible modifications related to patients with hormonal receptor (HR)–positive, human epidermal receptor 2 (HER2)–negative breast cancer, as well as patients with HER2-positive and triple negative breast cancer. The structured response formats used in this survey included binary (yes/no), nominal, and ordinal responses. Other options were also allowed, such as “I don’t know.”

### Testing of the Instrument

During pretesting and pilot testing, questions were reviewed by three breast cancer experts to check the consistency and appropriateness of the survey questions [16]. Then, the questions were reviewed by a nonexpert colleague to assess the dynamics, flow, and accessibility. Three medical oncologists performed pilot testing of the instrument.

We also conducted a clinical sensibility assessment to evaluate the comprehensiveness, clarity, and face validity of our instrument on a scale of 1 to 5. We invited 4 colleagues with methodologic and oncology expertise. The results of the clinical sensibility testing using mean scores on a 5-point scale suggested that the instrument had face validity (4.3), content validity (4.2), clarity (4.3), and discriminability (4.5). This survey was approved by the Institutional Review Board of King Abdullah Medical City, Makkah, Saudi Arabia (20-634).

### Study Procedures

We used a nonprobability snowball sampling design [17]. This web-based questionnaire was submitted to licensed medical oncologists involved in breast cancer management in Saudi Arabia, Egypt, and United Arab Emirates. We identified breast oncologists who are members of national oncology societies in the abovementioned countries through the databases of these societies. The oncologists were contacted by email to request their participation in the survey and were asked to send the survey link by email to other experienced breast oncologists. Two reminders were sent, 1 week apart, by email to the invited participants.

Participants received electronic links accompanied with concise instructions, the background and objectives of the survey, the target population, the expected time to finish the survey, and a request to participate voluntarily. They were required to register on the first page of the survey and provide their professional and academic degrees. Fellows or trainees were excluded, and only those respondents who had at least three years of experience in the management of breast cancer after completion of their specialist training were included. Participants consented to join the survey and to keep records of their professional details, institutes, and countries of clinical practice.

Each page of the survey contained 4 to 5 items, giving a total of 6 pages. The completeness of the survey was checked using JavaScript. To avoid duplicate entries, the survey could not be displayed again to the same user after their response was submitted. The anonymity of the answers was maintained using SurveyMonkey. The data were protected from unauthorized access. Only the authors and data analyst had access to the data.

### Outcome Assessment

The survey was conducted between July 10 and 30, 2020. We assessed the percentages of the responses of the breast oncologists. Descriptive statistics were used to summarize the data and report the views of the participants. We followed the CHERRIES (Checklist for Reporting Results of Internet E-Surveys) guidelines for conducting and reporting the results of the survey [18].

## Results

The survey was distributed to 100 people in Saudi Arabia, Egypt, and United Arab Emirates. A total of 82 people responded and agreed to participate in the survey. The completeness rate (completing all items of the survey) among the respondents was 100%.

### Characteristics of the Survey Participants

Of the 82 respondents, 62 (76%) were medical oncologists, while clinical oncologists and hematooncologists constituted 13 (16%) and 7 (9%) of the participants, respectively. The majority of respondents (72/82, 88%) worked in governmental hospitals, and 62% of the participants (51/82) had more than 10 years of work experience (Table 1).

**Table 1:** Characteristics of the survey participants (N=82).

Characteristic			Value, n (%)
**Country of practice**			
	Saudi Arabia	31 (38)	
	Egypt	39 (48)	
	United Arab Emirates	12 (15)	
**Subspecialty**			
	Medical oncologist		62 (76)
	Clinical oncologist		13 (16)
	Hematooncologist		7 (9)
**Duration of experience**			
	Less than 5 years		15 (18)
	5-10 years		16 (20)
	More than 10 years		51 (62)
**Type of institute of main practice**			
	Governmental hospital		72 (88)
	Academic institute		7 (9)
	Private hospital		3 (4)

### COVID-19 Prevalence and Requirement for Treatment Modifications

**Figure 1:**
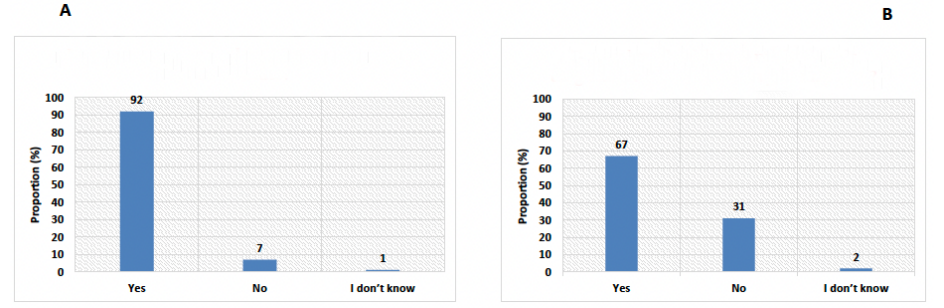
Responses to survey questions asking if the participants (A) have patients diagnosed with COVID-19 at their institute and (B) have health care professionals diagnosed with COVID-19 at their institute.

**Figure 2:**
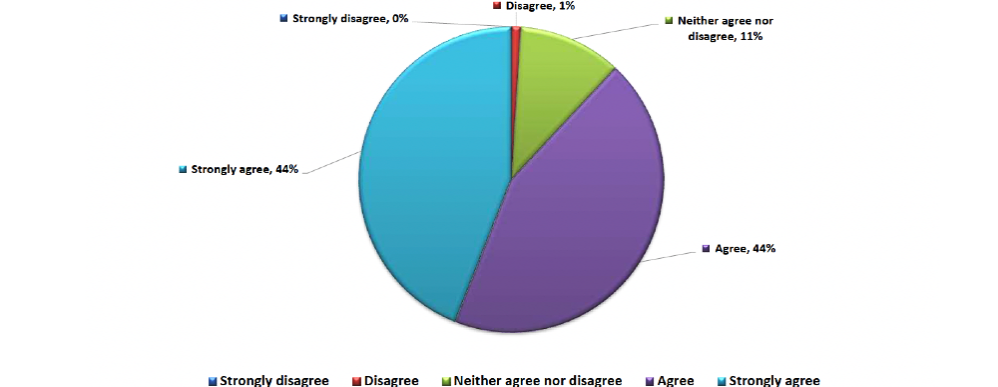
Participants' answers to the question of whether patients with cancer are at greater risk of COVID-19–related complications.

**Figure 3:**
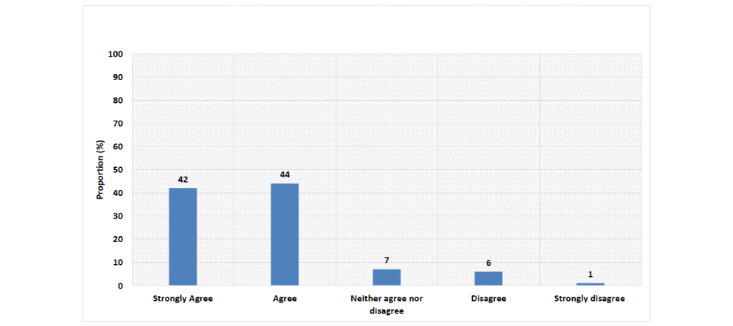
Participants' answers to the question of whether treatment modifications are required for patients with breast cancer during the COVID-19 pandemic.

The majority of the participants (75/82, 92%) reported that they had patients diagnosed with COVID-19 in their hospitals. Meanwhile, 67% (55/82) reported that HCPs had been diagnosed with COVID-19 in their institutes (Figure 1). Most of the respondents (72/82, 88%) agreed or strongly agreed that patients with cancer are at increased risk of COVID-19–related complications (Figure 2) and that the risk of these complications is different among patients with cancer (66/82, 81%) (Table 2). Noteworthily, the majority (70/82, 85%) supported modifications in breast cancer management during the COVID-19 pandemic (Figure 3). Similarly, the majority (76/82, 93%) endorsed the use of virtual multidisciplinary tumor boards for patients with breast cancer during the COVID-19 pandemic (Table 2).

**Table 2:** Responses to questions related to the risk of infection during the COVID-19 pandemic and required treatment modifications.

Question	Responses (N=82), n (%)				
	Strongly agree	Agree	Neither agree nor disagree	Disagree	Strongly disagree
Are patients with cancer at increased COVID-19 infection–related complications, such as respiratory failure?	36 (44)	36 (44)	9 (11)	1 (1)	0 (0)
Is the risk of serious complications of COVID-19 infection, such as respiratory failure, different among patients with cancer?	17 (21)	49 (60)	13 (16)	3 (4)	0 (0)
Are treatment modifications required for patients with breast cancer during the COVID-19 pandemic?	34 (42)	36 (44)	6 (7)	5 (6)	1 (1)
Is a virtual multidisciplinary approach for the management of patients with breast cancer mandatory in the current situation?	45 (55)	31 (38)	2 (2)	3 (4)	1 (1)

### Patients With HR-Positive Breast Cancer

#### Neoadjuvant Therapy

When neoadjuvant therapy is indicated, the majority of participants (61/82, 74%) supported using neoadjuvant hormonal therapy in selected patients (strong ER-positive, low Ki-67), while 11% (9/82) endorsed using neoadjuvant hormonal therapy in all patients. In T1/T2 tumors, when no downsizing is required, participants were divided over the use of neoadjuvant hormonal therapy as a bridge until the pandemic is over (Table 3).

**Table 3:** Suggested modifications to HR-positive, HER2-negative breast cancer practice for inpatient physicians.

Question and answer options			Responses (N=82), n (%)
**When neoadjuvant therapy is indicated (downsizing is required), what is the treatment of choice?**			
	Neoadjuvant chemotherapy		12 (15)
	Neoadjuvant hormonal therapy		9 (11)
	Neoadjuvant hormonal therapy in selected cases (strong estrogen receptor+, low Ki-67)		61 (74)
**Will neoadjuvant hormonal therapy be considered in T1 and T2 tumors (when no downsizing is required) as a bridge until the pandemic is over?**			
	Strongly agree	13 (16)	
	Strongly agree	34 (42)	
	Neither agree nor disagree	15 (18)	
	Disagree	19 (23)	
	Strongly disagree	1 (1)	
**Can adjuvant radiotherapy be given before adjuvant chemotherapy to avoid chemotherapy-induced neutropenia until the COVID-19 pandemic is over?**			
	Strongly agree	0 (0)	
	Agree	21 (26)	
	Neither agree nor disagree	16 (20)	
	Disagree	37 (45)	
	Strongly disagree	8 (10)	
**Using CDK 4/6^a^ inhibitors for new patients with metastatic HR^b^-positive, HER2^c^-negative breast cancer:**			
	CDK4/6 inhibitor+aromatase inhibitor is the treatment of choice		35 (43)
	Defer CDK 4/6 inhibitor to the second line until the pandemic is over in all cases		18 (22)
	Defer CDK 4/6 inhibitor to the second line until the pandemic is over in selected cases		29 (35)
**For new patients with nonvisceral metastasis, what is the treatment of choice in the first line during the COVID-19 pandemic ?**			
	Fulvestrant		9 (11)
	Aromatase inhibitor		45 (55)
	CDK 4/6 inhibitor+aromatase inhibitor		28 (34)
**For patients who have already started a CDK4/6 inhibitor+aromatase inhibitor, will the CDK4/6 inhibitor be held until the pandemic is over?**			
	Strongly agree	3 (4)	
	Agree	23 (28)	
	Neither agree nor disagree	20 (24)	
	Disagree	30 (37)	
	Strongly disagree	6 (7)	
**For patients with metastatic HR-positive, HER2-negative breast cancer, will you give everolimus or alpelisib in the second line?**			
	Strongly agree	3 (4)	
	Agree	23 (28)	
	Neither agree nor disagree	27 (33)	
	Disagree	27 (33)	
	Strongly disagree	2 (2)	
**For patients who have already started everolimus or alpelisib, will these medications be held until the pandemic is over?**			
	Strongly agree	4 (5)	
	Agree	18 (22)	
	Neither agree nor disagree	26 (32)	
	Disagree	33 (40)	
	Strongly disagree	1 (1)	

^a^CDK 4/6: cyclin-dependent kinases 4 and 6.

^b^HR: hormone receptor.

^c^HER2: human epidermal growth factor receptor 2.

#### Adjuvant Chemotherapy

When chemotherapy is indicated in early HR-positive, HER2-negative breast cancer, 58% (48/82) and 21% (17/82) of participants preferred giving 6 and 8 cycles, respectively, while 21% (17/82) reported that the number of chemotherapy cycles does not matter. Noteworthily, 55% (45/82) of the participants disagreed or strongly disagreed with delaying adjuvant chemotherapy until after finishing adjuvant radiotherapy, while only 26% (21/82) agreed with this approach (Table 3).

### Therapy for Patients With Metastasis

For metastatic patients, 43% of the participants (35/82) preferred using a CDK4/6 inhibitor with hormonal therapy in all patients, while 35% (29/82) preferred deferring CDK4/6 inhibitors to the second line in selected patients (Table 3). The treatments of choice of the survey participants for patients with nonvisceral metastasis were aromatase inhibitors (45/82, 55%), CDK4/6 inhibitor with aromatase inhibitor (28/82, 34%), and fulvestrant (9/82, 11%). For patients who had already started therapy with a CDK4/6 inhibitor, 44% of participants (36/82) disagreed or strongly disagreed with holding the CDK4/6 inhibitor until the pandemic is over, while only 32% (26/82) agreed or strongly agreed with that approach. Additionally, the participants were divided over the use of everolimus or alpelisib in second-line therapy. For patients who had already started therapy with everolimus, only 27% of participants (22/82) agreed or strongly agreed that everolimus should be held until the pandemic is over (Table 3).

### Patients With HER2-Positive Breast Cancer

Of the 82 participants, two-thirds (n=55, 67%) supported using adjuvant trastuzumab for 6 instead of 12 months in selected patients with HER2-positive breast cancer, such as low-risk patients, older patients, or patients with logistic barriers to receiving the medication during the COVID-19 pandemic.

For first-line treatment of metastatic HER2-positive, HR-positive breast cancer, 80% (66/82) of the participants supported the use of hormonal therapy with dual anti-HER2 blockade in selected patients (older persons, those with low tumor burden) (Table 4).

**Table 4:** Suggested treatment modifications in HER2-positive and triple-negative breast cancer.

Question and answer options			Responses (N=82), n (%)	
**Can adjuvant trastuzumab for 6 instead of 12 months can be considered in selected patients with HER2^a^-positive breast cancer (low-risk patients, older patients, or those with logistic barriers)?**				
	Strongly agree	17 (21)		
	Agree	38 (46)		
	Neither agree nor disagree	7 (9)		
	Disagree	17 (21)		
	Strongly disagree	3 (4)		
**For first line treatment of metastatic HER2-positive, HR^b^-positive breast cancer, will hormonal therapy with dual anti-HER2 blockade be considered in selected patients (older patients, those with low tumor burden)?**				
	Strongly agree	13 (16)		
	Agree	53 (65)		
	Neither agree nor disagree	7 (9)		
	Disagree	8 (10)		
	Strongly disagree	1 (1)		
**In metastatic triple negative breast cancer with *BRCA* mutation and *PD-L1*^c^ <1%, what is the first-line treatment of choice?**				
	PARP^d^ inhibitor			34 (41)
	Platinum-based chemotherapy			30 (37)
	Taxanes			11 (13)
	Other			7 (9)
**In metastatic triple negative breast cancer with *BRCA* mutation and *PD-L1* >1%, what is the first-line treatment of choice?**				
	PARP^d^ inhibitor			14 (17)
	Atezolizumab+nab-paclitaxel			58 (71)
	Taxanes			5 (6)
	Other			5 (6)
**When chemotherapy is indicated for patients with metastatic breast cancer, if intravenous chemotherapy is chosen, what is the preferred regimen?**				
	Taxane: 3-weekly regimen	49 (60)		
	Taxane: weekly regimen	17 (21)		
	Anthracycline	9 (11)		
	Gemcitabine	4 (5)		
	Vinorelbine	3 (4)		

^a^HER2: human epidermal growth factor receptor 2.

^b^HR: hormone receptor.

^c^*PD-L1*: programmed cell death 1 ligand 1.

^d^PARP: poly–(adenosine diphosphate–ripose) polymerase.

### Patients With Triple-Negative Breast Cancer

Regarding the choice of first-line treatment in metastatic patients with *BRCA* mutation and programmed cell death 1 ligand 1 (*PD-L1*) <1%, the preferred treatment choices were poly–(adenosine diphosphate–ripose) polymerase (PARP) inhibitors (34/82, 41%), platinum-based chemotherapy (30/82, 37%), and taxanes (11/82, 13%). Meanwhile, in metastatic triple-negative breast cancer with *BRCA* mutation and *PD-L1* >1%, atezolizumab with nab-paclitaxel was the preferred choice for 71% (58/82) of the participants. When chemotherapy is indicated for patients with metastatic breast cancer, participants were divided between oral (39/82, 48%) and intravenous (IV) (43/82, 52%) chemotherapy. If IV chemotherapy was chosen, the preferred choices of the survey participants were 3-weekly taxane (49/82, 60%) and weekly taxane (17/82, 21%) (Table 4). During the COVID-19 pandemic, 52% (43/82) of participants supported lowering the threshold of prescription of granulocyte colony-stimulating factor following chemotherapy.

## Discussion

### Principal Findings

In this survey, we explored the views of breast cancer oncologists practicing in three Middle Eastern countries regarding modifications in breast cancer management during the COVID-19 pandemic. The majority of the participants reported having COVID-19 cases in their institutes and believed that treatment modifications were required during the pandemic. We focused on modifications related to systemic therapy of patients with breast cancer, and these were categorized according to different breast cancer subtypes. The majority of participants supported using treatment strategies that decreased the risk of COVID-19 infection–related complications, such as using neoadjuvant hormonal therapy in patients with HR-positive/HER2 negative breast cancer, using 6 months of adjuvant trastuzumab in selected patients with HER2-positive disease, and using hormonal therapy with dual anti-HER2 blockade in metastatic HR-positive/HER2-positive patients. Meanwhile, participants were divided over some suggested modifications, such as using IV versus oral chemotherapy in metastatic patients when indicated.

Patients with cancer are at increased risk for severe disease and increased mortality due to COVID-19 infection [19]. In hospitalized patients with COVID-19, case fatality rates reported among patients with cancer are higher compared to those of other patients (29.4% vs 10.2%, respectively; *P*<.001) [20]. Large cohort studies have consistently demonstrated that all-cause mortality and the likelihood of ICU admission are higher in patients with cancer, even after adjustment for age, sex, diabetes, smoking, cardiovascular and pulmonary disease, and other common risk factors for COVID-19 severity [20-22]. These data highlight the critical need to decrease the risk of COVID-19 infection among patients with cancer.

Therefore, management of patients with breast cancer is challenging during the COVID-19 pandemic given the limitations of access to care, maintaining the level of patient care, travel restrictions, and immune suppression secondary to therapeutic modalities or the disease itself. This highlights the importance of the abovementioned modifications to breast cancer management to decrease the risk of myelosuppression/immune suppression and decrease the frequency of hospital visits and need of laboratory monitoring in addition to adopting alternative strategies when standard treatment approaches cannot be provided. Here, we will explore the scientific evidence for the different survey items supported by the participating oncologists.

### CDK 4/6 Inhibitors in HR-Positive, HER2-Negative Breast Cancer

CDK 4/6 inhibitors with an aromatase inhibitor are currently the standard first-line therapy in HR-positive, HER2-negative patients without visceral crisis. Several clinical trials have established the survival benefit of these medications [7-9]. Neutropenia is the most frequent side effect encountered with this class of medications [7-9]. This may pose a particular risk in the era of the COVID-19 pandemic, particularly in older patients and those with low baseline neutrophil count. Moreover, in September 2019, the US Food and Drug Administration released a warning of rare but serious drug-induced interstitial pneumonitis with CDK 4/6 inhibitors [23]. Therefore, delaying CDK 4/6 inhibitors to second-line therapy until the pandemic is over may be an appropriate strategy, given that they demonstrated survival benefit in the second line when added to fulvestrant [24,25]. Noteworthily, ESMO recommendations reported that postponing the incorporation of a CDK4/6 inhibitor in the first line for patients presenting with special patterns of disease (eg, bone only, low burden, de novo metastatic disease) could be an option, especially in the older population [10].

Interestingly, in the FALCON study, progression-free survival (PFS) was significantly improved with fulvestrant monotherapy compared to anastrozole as a first-line therapy in patients with nonvisceral metastasis (22.3 vs 13.8 months, respectively), which makes fulvestrant an attractive first-line option that is recommended for this category of patients [26].

### mTOR and PIK3 Inhibitors

Everolimus and alpelisib improved PFS when added to hormonal therapy in the BOLERO2 and SOLAR1 studies, respectively [10,11]. However, these medications are associated with adverse events such as hyperglycemia and noninfectious pneumonitis; therefore, their use may be problematic in the current era [27,28]. Patients with noninfectious pneumonitis may have similar manifestations to those of COVID-19 infection, such as dyspnea, cough, hypoxia, and fever, thereby complicating the diagnosis, and they may exacerbate potential respiratory drawbacks of COVID-19 infection. Noteworthily, treatment with steroids is required in patients with grade ≥2 noninfectious pneumonitis, which may put patients at increased risk of COVID-19 infection [29]. ESMO advises that the addition of mTOR or *PI3KCA* inhibitors is not of immediate priority and should be avoided [10].

### Neoadjuvant Hormonal Therapy in HR-Positive, HER2-Negative Breast Cancer

Several trials have investigated the use of neoadjuvant hormonal therapy in postmenopausal patients with bulky HR-positive, HER2-negative disease to achieve better surgical outcomes. Several studies and meta-analyses demonstrated improved rates of breast conservative surgery with aromatase inhibitors compared to tamoxifen [30-33]. Data from randomized trials in postmenopausal patients displayed that higher ER and lower Ki-67 levels were signiﬁcantly correlated with a higher probability of response [31,34]. Therefore, neoadjuvant hormonal therapy can be a good strategy to postpone breast surgery without compromising patients’ outcome, with the current limitations in health services with limited surgical slots. Noteworthily, neoadjuvant endocrine therapy is recommended by ESMO as an option for patients with ER-positive/HER2-negative breast cancer to enable deferral of surgery by 6 to 12 months in clinical stage I or II breast cancers [10].

### Choice of Systemic Chemotherapy in Metastatic Breast Cancer in the COVID-19 Era

Oral chemotherapeutic agents, including capecitabine and vinorelbine, display activity in heavily pretreated patients; they have demonstrated overall response rates of up to 35% to 40%, which may be comparable to those of anthracyclines and taxanes [35-39]. Oral chemotherapy may be more convenient in the COVID-19 era. Generally, these agents are well tolerated and can be dispensed for several cycles and delivered to patients via medication delivery services. This approach can limit hospital visits and exposure to infection.

### HR-Positive, HER2-Positive Breast Cancer: Chemotherapy-Free Regimens

Treatment with hormonal therapy combined with dual anti-HER2 therapy in HER2–positive/HR-positive MBC was assessed in several trials with encouraging results [40-42]. This strategy can be considered in selected patients, such as older patients, patients with borderline performance status, and patients with limited tumor burden. This chemotherapy-free approach can avoid neutropenia and other chemotherapy-related adverse events to minimize possible COVID-19–associated sequelae.

### Duration of Adjuvant Trastuzumab in HER2-Positive Breast Cancer

Several studies assessed adjuvant trastuzumab for 6 versus 12 months, including the Hellenic Oncology Research Group, PHARE, and PERSEPHONE studies [43-45]. All studies, except for the PERSEPHONE study, failed to demonstrate noninferiority of shorter versus longer duration of adjuvant trastuzumab. Meanwhile, the absolute difference in survival was 2% on average [46]. These data may be reassuring because in certain groups of patients, particularly those with low risk of relapse and logistic limitations, the survival outcome will not be greatly compromised if the adjuvant trastuzumab duration is limited to 6 months. Noteworthily, for selected patients with HER2-positive breast cancer, such as low-risk patients or older patients with cardiovascular or other comorbidities, adjuvant anti-HER2 therapy may reasonably be discontinued after 6 months instead of 12 months of treatment according to ESMO recommendations during the COVID-19 pandemic [10].

However, our study has some limitations. This survey was conducted in 3 Middle Eastern countries, which may not reflect current practice in other parts of the world. Furthermore, the sample size is relatively small, which is mostly related to the fact that many oncologists in the region are general oncologists without specific practice in breast cancer. In addition, differences in economic status, availability of medications and medication delivery services, and health system infrastructure may affect the application of the abovementioned modification strategies.

Finally, these modifications need to be discussed on a local basis, taking into account the local infrastructure and available resources. In addition, virtual tumor board discussion is critically important in this context to choose the most convenient therapeutic strategy without compromising treatment efficacy or patient safety.

## Abbreviations

CDK4/6: cyclin-dependent kinases 4 and 6
CHERRIES: Checklist for Reporting Results of Internet E-Surveys
ESMO: European Society of Medical Oncology
HCP: health care professional
HER2: human epidermal growth factor receptor 2
HR: hormone receptor
ICU: intensive care unit
IV: intravenous
PARP: poly–(adenosine diphosphate–ripose) polymerase
PFS: progression-free survival
*PD-L1*: programmed cell death 1 ligand 1

## References

[1] Hanna TP, Evans GA, Booth CM (2020). Cancer, COVID-19 and the precautionary principle: prioritizing treatment during a global pandemic. Nat Rev Clin Oncol.

[2] Liang W, Guan W, Chen R, Wang W, Li J, Xu K, Li C, Ai Q, Lu W, Liang H, Li S, He J (2020). Cancer patients in SARS-CoV-2 infection: a nationwide analysis in China. Lancet Oncol.

[3] Brunetti O, Derakhshani A, Baradaran B, Galvano A, Russo A, Silvestris N (2020). COVID-19 infection in cancer patients: how can oncologists deal with these patients?. Front Oncol.

[4] Di Lorenzo G, Di Trolio R (2020). Coronavirus disease (COVID-19) in Italy: analysis of risk factors and proposed remedial measures. Front Med (Lausanne).

[5] Lambertini M, Toss A, Passaro A, Criscitiello Carmen, Cremolini Chiara, Cardone Claudia, Loupakis Fotios, Viscardi Giuseppe, Meattini Icro, Dieci Maria Vittoria, Ferrara Roberto, Giusti Raffaele, Maio Massimo Di (2020). Cancer care during the spread of coronavirus disease 2019 (COVID-19) in Italy: young oncologists' perspective. ESMO Open.

[6] Carrick S, Parker S, Thornton C, Ghersi D, Simes J, Wilcken N (2005). Single agent versus combination chemotherapy for metastatic breast cancer. Cochrane Database Syst Rev.

[7] Finn RS, Martin M, Rugo HS, Jones S, Im S, Gelmon K, Harbeck N, Lipatov ON, Walshe JM, Moulder S, Gauthier E, Lu DR, Randolph S, Diéras V, Slamon DJ (2016). Palbociclib and letrozole in advanced breast cancer. N Engl J Med.

[8] O'Shaughnessy Joyce, Petrakova K, Sonke GS, Conte P, Arteaga CL, Cameron DA, Hart LL, Villanueva C, Jakobsen E, Beck JT, Lindquist D, Souami F, Mondal S, Germa C, Hortobagyi GN (2018). Ribociclib plus letrozole versus letrozole alone in patients with de novo HR+, HER2- advanced breast cancer in the randomized MONALEESA-2 trial. Breast Cancer Res Treat.

[9] Goetz MP, Toi M, Campone M, Sohn J, Paluch-Shimon S, Huober J, Park IH, Trédan O, Chen S, Manso L, Freedman OC, Garnica Jaliffe G, Forrester T, Frenzel M, Barriga S, Smith IC, Bourayou N, Di Leo A (2017). MONARCH 3: abemaciclib as initial therapy for advanced breast cancer. JCO.

[10] ESMO Clinical Practice Guidelines: Breast Cancer. European Society of Medical Oncology.

[11] (2020). COVID-19 guidelines for triage of breast cancer patients. American College of Surgeons.

[12] COVID-19 Resources. National Comprehensive Cancer Network.

[13] (2020). Pandemic planning clinical guideline for patients with cancer. Cancer Care Ontario.

[14] Méndez LA (2017). From Lisp to FuzzyLisp. Studies in Fuzziness and Soft Computing, vol 349.

[15] Passmore C, Dobbie Alison E, Parchman Michael, Tysinger James (2002). Guidelines for constructing a survey. Fam Med.

[16] Collins D (2003). Pretesting survey instruments: an overview of cognitive methods. Qual Life Res.

[17] Aday LA, Cornelius LJ (2006). Designing and Conducting Health Surveys: A Comprehensive Guide.

[18] Eysenbach G (2004). Improving the quality of web surveys: the Checklist for Reporting Results of Internet E-Surveys (CHERRIES). J Med Internet Res.

[19] Fung M, Babik J (2021). COVID-19 in immunocompromised hosts: what we know so far. Clin Infect Dis.

[20] Meng Y, Lu W, Guo E, Liu J, Yang B, Wu P, Lin S, Peng T, Fu Y, Li F, Wang Z, Li Y, Xiao R, Liu C, Huang Y, Lu F, Wu X, You L, Ma D, Sun C, Wu P, Chen G (2020). Cancer history is an independent risk factor for mortality in hospitalized COVID-19 patients: a propensity score-matched analysis. J Hematol Oncol.

[21] Dai M, Liu D, Liu M, Zhou F, Li G, Chen Z, Zhang Z, You H, Wu M, Zheng Q, Xiong Y, Xiong H, Wang C, Chen C, Xiong F, Zhang Y, Peng Y, Ge S, Zhen B, Yu T, Wang L, Wang H, Liu Y, Chen Y, Mei J, Gao X, Li Z, Gan L, He C, Li Z, Shi Y, Qi Y, Yang J, Tenen DG, Chai L, Mucci LA, Santillana M, Cai H (2020). Patients with cancer appear more vulnerable to SARS-COV-2: a multi-center study during the COVID-19 outbreak. Cancer Discov.

[22] Tian J, Yuan X, Xiao J, Zhong Q, Yang C, Liu B, Cai Y, Lu Z, Wang J, Wang Y, Liu S, Cheng B, Wang J, Zhang M, Wang L, Niu S, Yao Z, Deng X, Zhou F, Wei W, Li Q, Chen X, Chen W, Yang Q, Wu S, Fan J, Shu B, Hu Z, Wang S, Yang X, Liu W, Miao X, Wang Z (2020). Clinical characteristics and risk factors associated with COVID-19 disease severity in patients with cancer in Wuhan, China: a multicentre, retrospective, cohort study. Lancet Oncol.

[23] Astor L (2019). FDA warns of lung inflammation from CDK4/6 inhibitor use in breast cancers. Targeted Oncology.

[24] Cristofanilli M, Turner NC, Bondarenko I, Ro J, Im S, Masuda N, Colleoni M, DeMichele A, Loi S, Verma S, Iwata H, Harbeck N, Zhang K, Theall KP, Jiang Y, Bartlett CH, Koehler M, Slamon D (2016). Fulvestrant plus palbociclib versus fulvestrant plus placebo for treatment of hormone-receptor-positive, HER2-negative metastatic breast cancer that progressed on previous endocrine therapy (PALOMA-3): final analysis of the multicentre, double-blind, phase 3 randomised controlled trial. Lancet Oncol.

[25] Sledge GW, Toi M, Neven P, Sohn J, Inoue K, Pivot X, Burdaeva O, Okera M, Masuda N, Kaufman PA, Koh H, Grischke E, Frenzel M, Lin Y, Barriga S, Smith IC, Bourayou N, Llombart-Cussac A (2017). MONARCH 2: abemaciclib in combination with fulvestrant in women with HR+/HER2− advanced breast cancer who had progressed while receiving endocrine therapy. JCO.

[26] Robertson JFR, Bondarenko IM, Trishkina E, Dvorkin M, Panasci L, Manikhas A, Shparyk Y, Cardona-Huerta S, Cheung K, Philco-Salas MJ, Ruiz-Borrego M, Shao Z, Noguchi S, Rowbottom J, Stuart M, Grinsted LM, Fazal M, Ellis MJ (2016). Fulvestrant 500 mg versus anastrozole 1 mg for hormone receptor-positive advanced breast cancer (FALCON): an international, randomised, double-blind, phase 3 trial. Lancet.

[27] Baselga J, Campone M, Piccart M, Burris HA, Rugo HS, Sahmoud T, Noguchi S, Gnant M, Pritchard KI, Lebrun F, Beck JT, Ito Y, Yardley D, Deleu I, Perez A, Bachelot T, Vittori L, Xu Z, Mukhopadhyay P, Lebwohl D, Hortobagyi GN (2012). Everolimus in postmenopausal hormone-receptor–positive advanced breast cancer. N Engl J Med.

[28] André F, Ciruelos E, Rubovszky G, Campone M, Loibl S, Rugo HS, Iwata H, Conte P, Mayer IA, Kaufman B, Yamashita T, Lu Y, Inoue K, Takahashi M, Pápai Z, Longin A, Mills D, Wilke C, Hirawat S, Juric D (2019). Alpelisib for PIK3CA-mutated, hormone receptor–positive advanced breast cancer. N Engl J Med.

[29] Cazzaniga ME, Danesi R, Girmenia C, Invernizzi P, Elvevi A, Uguccioni M, NetworkER+ (2019). Management of toxicities associated with targeted therapies for HR-positive metastatic breast cancer: a multidisciplinary approach is the key to success. Breast Cancer Res Treat.

[30] Eiermann W, Paepke S, Appfelstaedt J, Llombart-Cussac A, Eremin J, Vinholes J, Mauriac L, Ellis M, Lassus M, Chaudri-Ross H, Dugan M, Borgs M, Semiglazov V (2001). Preoperative treatment of postmenopausal breast cancer patients with letrozole: a randomized double-blind multicenter study. Ann Oncol.

[31] Smith IE, Dowsett M, Ebbs SR, Dixon JM, Skene A, Blohmer J, Ashley SE, Francis S, Boeddinghaus I, Walsh G (2005). Neoadjuvant treatment of postmenopausal breast cancer with anastrozole, tamoxifen, or Both in Combination: The Immediate Preoperative anastrozole, tamoxifen, or combined with tamoxifen (IMPACT) multicenter double-blind randomized trial. J Clin Oncol.

[32] Cataliotti L, Buzdar AU, Noguchi S, Bines J, Takatsuka Y, Petrakova K, Dube P, de Oliveira Celia Tosello (2006). Comparison of anastrozole versus tamoxifen as preoperative therapy in postmenopausal women with hormone receptor-positive breast cancer: the Pre-Operative "Arimidex" Compared to Tamoxifen (PROACT) trial. Cancer.

[33] Seo JH, Kim YH, Kim JS (2009). Meta-analysis of pre-operative aromatase inhibitor versus tamoxifen in postmenopausal woman with hormone receptor-positive breast cancer. Cancer Chemother Pharmacol.

[34] Ellis MJ, Coop A, Singh B, Mauriac L, Llombert-Cussac A, Jänicke F, Miller WR, Evans DB, Dugan M, Brady C, Quebe-Fehling E, Borgs M (2001). Letrozole is more effective neoadjuvant endocrine therapy than tamoxifen for ErbB-1– and/or ErbB-2–positive, estrogen receptor–positive primary breast cancer: evidence from a Phase III randomized trial. J Clin Oncol.

[35] Blum JL, Dieras V, Lo Russo PM, Horton J, Rutman O, Buzdar A, Osterwalder B (2001). Multicenter, Phase II study of capecitabine in taxane-pretreated metastatic breast carcinoma patients. Cancer.

[36] Venturini M, Paridaens R, Rossner D, Vaslamatzis M, Nortier J, Salzberg M, Rodrigues H, Bell R (2007). An open-label, multicenter study of outpatient capecitabine monotherapy in 631 patients with pretreated advanced breast cancer. Oncology.

[37] Fumoleau P, Delgado FM, Delozier T, Monnier A, Gil Delgado MA, Kerbrat P, Garcia-Giralt E, Keiling R, Namer M, Closon MT (1993). Phase II trial of weekly intravenous vinorelbine in first-line advanced breast cancer chemotherapy. J Clin Oncol.

[38] Vogel C, O’Rourke M, Winer E, Hochster H, Chang A, Adamkiewicz B, White R, McGuirt C (1999). Vinorelbine as first-line chemotherapy for advanced breast cancer in women 60 years of age or older. Ann Oncol.

[39] Romero A, Rabinovich MG, Vallejo CT, Perez JE, Rodriguez R, Cuevas MA, Machiavelli M, Lacava JA, Langhi M, Romero Acuña L (1994). Vinorelbine as first-line chemotherapy for metastatic breast carcinoma. J Clin Oncol.

[40] Rimawi M, Ferrero J, de la Haba-Rodriguez J, Poole C, De Placido S, Osborne CK, Hegg R, Easton V, Wohlfarth C, Arpino G (2018). First-line trastuzumab plus an aromatase inhibitor, with or without pertuzumab, in human epidermal growth factor receptor 2–positive and hormone receptor–positive metastatic or locally advanced breast cancer (PERTAIN): a randomized, open-label Phase II trial. J Clin Oncol.

[41] Johnston S, Hegg R, Im S, Park Ih, Burdaeva O, Kurteva G, Press Mf, Tjulandin S, Iwata H, Simon Sd, Kenny S, Sarp S, Izquierdo Ma, Williams Ls, Gradishar Wj (2018). Phase III, randomized study of dual human epidermal growth factor receptor 2 (HER2) blockade with lapatinib plus trastuzumab in combination with an aromatase inhibitor in postmenopausal women with HER2-positive, hormone receptor-positive metastatic breast cancer: ALTERNATIVE. J Clin Oncol.

[42] Johnston S, Pippen J, Pivot X, Lichinitser M, Sadeghi S, Dieras V, Gomez HL, Romieu G, Manikhas A, Kennedy MJ, Press MF, Maltzman J, Florance A, O'Rourke L, Oliva C, Stein S, Pegram M (2009). Lapatinib combined with letrozole versus letrozole and placebo as first-line therapy for postmenopausal hormone receptor–positive metastatic breast cancer. J Clin Oncol.

[43] Mavroudis D, Saloustros E, Malamos N, Kakolyris S, Boukovinas I, Papakotoulas P, Kentepozidis N, Ziras N, Georgoulias V, Breast Cancer Investigators of Hellenic Oncology Research Group (HORG)‚ Athens‚ Greece (2015). Six versus 12 months of adjuvant trastuzumab in combination with dose-dense chemotherapy for women with HER2-positive breast cancer: a multicenter randomized study by the Hellenic Oncology Research Group (HORG). Ann Oncol.

[44] Pivot X, Romieu G, Debled M, Pierga J, Kerbrat P, Bachelot T, Lortholary A, Espié M, Fumoleau P, Serin D, Jacquin J, Jouannaud C, Rios M, Abadie-Lacourtoisie S, Tubiana-Mathieu N, Cany L, Catala S, Khayat D, Pauporté I, Kramar A (2013). 6 months versus 12 months of adjuvant trastuzumab for patients with HER2-positive early breast cancer (PHARE): a randomised phase 3 trial. Lancet Oncol.

[45] Earl HM, Hiller L, Vallier A, Loi S, Howe D, Higgins HB, McAdam K, Hughes-Davies L, Harnett AN, Ah-See M, Simcock R, Rea DW, Mansi J, Abraham J, Caldas C, Hulme C, Miles D, Wardley AM, Cameron DA, Dunn J (2018). PERSEPHONE: 6 versus 12 months (m) of adjuvant trastuzumab in patients (pts) with HER2 positive (+) early breast cancer (EBC): Randomised phase 3 non-inferiority trial with definitive 4-year (yr) disease-free survival (DFS) results. J Clin Oncol.

[46] Hurvitz SA (2019). Is the duration of adjuvant trastuzumab debate still clinically relevant?. Lancet.

